# Moments of derivatives of quadratic Dirichlet *L*-functions with prime conductor

**DOI:** 10.1007/s40993-026-00704-7

**Published:** 2026-02-07

**Authors:** Christopher G. Best

**Affiliations:** Department of Mathematics, University of Exeter, Exeter, EX4 4QF United Kingdom

**Keywords:** Mixed moments, Derivatives of Dirichlet *L*-functions, Function fields, Primary 11M06, Secondary 11M38, 11M50

## Abstract

We compute an asymptotic formula for the mixed second moment of the $\mu$-th and $\nu$-th derivatives of quadratic Dirichlet *L*-functions over monic, irreducible polynomials in the function field setting.

## Introduction

The moments of various families of *L*-functions play a central role in analytic number theory and it is a long-standing problem to study their asymptotic behaviour. For instance, the 2*k*-th moment of the Riemann zeta function on the critical line is given by

$$\begin{aligned} M_k(T)=\frac{1}{T} \int _1^T |\zeta (\tfrac{1}{2}+it)|^{2k} \, dt, \end{aligned}$$

with the goal being to determine an asymptotic for $M_k(T)$ as $T \rightarrow \infty$. Currently, formulae are known only for the second and fourth moments due to Hardy and Littlewood [22] and Ingham [23], respectively. Explicitly, one has

$$\begin{aligned} M_1(T) \sim \log T, \end{aligned}$$

and

$$\begin{aligned} M_2(T) \sim \frac{1}{2 \pi ^2} \log ^4 T. \end{aligned}$$

The long-standing conjecture for the 2*k*-th moment is that as $T \rightarrow \infty$,

1.1 $$\begin{aligned} M_k(T) \sim a_k g_k (\log T)^{k^2}, \end{aligned}$$

where $a_k$ is a well understood arithmetic factor in the form of an Euler product, and where $g_k$ is some geometric factor. In their breakthrough work [28], Keating and Snaith put forward an explicit expression for $g_k$ using random matrix theory. The heuristic ‘recipe’ of Conrey et al. [16] was used to formulate a conjecture for the integral moments of the zeta function which predicts that up to a power saving error,

$$\begin{aligned} M_k(T) \sim P_k(\log T), \end{aligned}$$

with $P_k(x)$ an explicit polynomial of degree $k^2$.

In [23], Ingham also considered a mixed second moment of derivatives of the zeta function and for integers $\mu ,\nu \ge 0$, obtained the asymptotic formula

$$\begin{aligned} \frac{1}{T} \int _1^T \zeta ^{(\mu )}(\tfrac{1}{2}+it) \zeta ^{(\nu )}(\tfrac{1}{2}-it) \, dt \sim \frac{1}{\mu +\nu +1} (\log T)^{\mu +\nu +1}. \end{aligned}$$

Since $\zeta ^{(\mu )}(\tfrac{1}{2}-it)=\overline{\zeta ^{(\mu )}(\tfrac{1}{2}+it)}$, Ingham’s result implies that

$$\begin{aligned} \frac{1}{T} \int _1^T |\zeta ^{(\mu )}(\tfrac{1}{2}+it)|^2 \, dt \sim \frac{1}{2\mu +1} (\log T)^{2\mu +1}. \end{aligned}$$

The fourth moment of the derivative of the zeta function was considered by Conrey [15] who showed that

$$\begin{aligned} \frac{1}{T} \int _1^T |\zeta '(\tfrac{1}{2}+it)|^4 \, dt \sim \frac{61}{1680 \pi ^2} \left( \log \frac{T}{2\pi } \right) ^8. \end{aligned}$$

Additionally, Conrey proved that as $m \rightarrow \infty$,

$$\begin{aligned} \frac{\pi ^2}{6} C_{2,m} \sim \frac{1}{16 m^4}, \end{aligned}$$

where

$$\begin{aligned} C_{k,m}:=\lim _{T \rightarrow \infty } T^{-1} \left( \log \frac{T}{2\pi } \right) ^{-k^2-2km} \int _1^T |\zeta ^{(m)}(\tfrac{1}{2}+it)|^{2k} \, dt. \end{aligned}$$

Random matrix theory serves as a very useful tool for formulating conjectures on the analytic properties of the Riemann zeta function and other *L*-functions. In particular, it is now well known that *L*-functions can be modelled by the characteristic polynomials of random matrices. In this direction, Conrey, Rubinstein and Snaith [17] studied the moments of the derivative of characteristic polynomials $\Lambda _A(s)$ of matrices $A \in U(N)$ chosen from the Circular Unitary Ensemble (CUE). They proved that as $N \rightarrow \infty$ with $k \in \mathbb {N}$,

$$\begin{aligned} \int _{U(N)} |\Lambda _A'(1)|^{2k} \, dA \sim b_k N^{k^2+2k}, \end{aligned}$$

where *dA* denotes the normalised Haar measure on the group of unitary matrices *U*(*N*), and where $b_k$ has an explicit expression in terms of a determinant involving modified Bessel functions of the first kind. Their result led them to then conjecture that as $T \rightarrow \infty$,

$$\begin{aligned} \int _1^T |\zeta '(\tfrac{1}{2}+it)|^{2k} \, dt \sim a_k b_k (\log T)^{k^2+2k}, \end{aligned}$$

where $a_k$ is the same arithmetic factor appearing in the conjectural formula (1.1) for the moments of the zeta function itself. Recently, Keating and Wei [29] have obtained asymptotic formulae for the moments and joint moments of arbitrary order derivatives of the characteristic polynomials of random unitary matrices. This allowed them to put forward conjectures for the joint moments of higher order derivatives of the Riemann zeta function and of derivatives of Hardy’s *Z*-function.

One family of *L*-functions whose moments have received a considerable amount of attention is the quadratic Dirichlet *L*-functions $L(s,\chi _d)$. For a fundamental discriminant *d*, the quadratic character $\chi _d=(\tfrac{d}{n})$ is defined by the Kronecker symbol and $L(s,\chi _d)$ is the associated *L*-function. In this case, the *k*-th moment is conjectured to be given asymptotically by

1.2 $$\begin{aligned} \frac{1}{X} \mathop {{\mathop {\sum }\nolimits ^*}}\limits _{0<d \le X} L(\tfrac{1}{2},\chi _d)^k \sim c_k (\log X)^{k(k+1)/2}, \end{aligned}$$

with an explicit expression for $c_k$ put forward by Keating and Snaith [27], again using random matrix theory. Previously, asymptotic formulae for the first and second moments of this family were obtained by Jutila [26]. Restricting to discriminants of the form 8*d* where *d* is an odd, square-free integer, Soundararajan [37] used a Poisson summation formula to obtain both the second and third moments with a power saving error term. Recently, assuming the generalised Riemann hypothesis (GRH), Shen [35] proved an asymptotic formula for the fourth moment congruent with the prediction of (1.2). Subsequently, Shen and Stucky [36] were able to remove the assumption of GRH from the main result of [35] and obtain the next three lower order terms in the fourth moment.

A similar problem is to study the moments of these quadratic *L*-functions over primes rather than over discriminants. Defining the quadratic character $\chi _p(n)=(\tfrac{n}{p})$ using the Legendre symbol for a prime *p*, one now considers the *k*-th moment

$$\begin{aligned} \frac{1}{X} \sum _{p \le X} L(\tfrac{1}{2},\chi _p)^k. \end{aligned}$$

The problem is considerably more difficult over primes and as such, only the first moment has been computed unconditionally by Jutila [26]. Sharp upper and lower bounds for the second moment were obtained by Baluyot and Pratt in [11], who further obtained an asymptotic formula for the second moment under the assumption of GRH. A similar conjecture to (1.2) is believed to hold for these moments with a corresponding leading coefficient since both families have symplectic symmetry type.

Another related family is that of quadratic twists of a modular *L*-function. This family is of particular interest when the modular *L*-function is attached to an elliptic curve (and so the twisted *L*-functions are attached to the quadratic twists of that curve) as according to the Birch and Swinnerton-Dyer conjecture, the order of vanishing of these *L*-functions at the central point is equal to the rank of their associated curve. Given this arithmetic motivation, the first moment of the derivative of these *L*-functions was studied in [14, 24, 31]. Assuming GRH, an asymptotic formula for the second moment of this family was obtained by Soundararajan and Young [38], with their result then being made unconditional by Li in [30]. Restricting to the half of this family whose sign of their functional equation is $-1$, Petrow [32] proved a number of asymptotic formulae on the first and second moments of the derivative of these *L*-functions.

In this paper, we consider moments of derivatives of quadratic Dirichlet *L*-functions with prime conductor in the function field setting. Over function fields, the moments of quadratic Dirichlet *L*-functions have also been extensively studied, with ideas and advancements in one setting regularly being translated to yield progress in the other. Analogously to the number field setting, one seeks asymptotic formulae as $g \rightarrow \infty$ for the *k*-th moment

$$\begin{aligned} \frac{1}{|\mathcal {H}_{2g+1}|} \sum _{D \in \mathcal {H}_{2g+1}} L(\tfrac{1}{2},\chi _D)^k, \end{aligned}$$

where $\mathcal {H}_{2g+1}$ denotes the set of monic, square-free polynomials of degree $2g+1$ in $\mathbb {F}_q[t]$, also known as the hyperelliptic ensemble. The first moment was computed by Andrade and Keating [6] with the main term being of size *g*. By applying a Poisson summation formula over $\mathbb {F}_q[t]$, Florea [21] proved the existence of a secondary main term of size $q^{-4g/3} g$ in the first moment. In [20], Florea then used this approach to evaluate the second and third moments, obtaining a power saving error in both cases. The fourth moment was also considered by Florea [19] who proved an asymptotic formula with the first three main terms. A general conjecture for the integral *k*-th moments, including lower order terms, was formulated by Andrade and Keating [8] by adapting the recipe of [16] to the function field setting.

As before, it is also interesting to study the moments of quadratic Dirichlet *L*-functions over prime (monic and irreducible) polynomials in $\mathbb {F}_q[t]$. Denoting the set of monic, irreducible polynomials of degree $2g+1$ by $\mathcal {P}_{2g+1}$, the *k*-th moment of this family

$$\begin{aligned} \frac{1}{|\mathcal {P}_{2g+1}|} \sum _{P \in \mathcal {P}_{2g+1}} L(\tfrac{1}{2},\chi _P)^k \end{aligned}$$

is more challenging to evaluate than in the case over square-free polynomials, similarly to the number field setting. Asymptotic formulae for the first and second moment were computed by Andrade and Keating [7] with the main terms being of size *g* and $g^3$, respectively. In [12], Bui and Florea obtained a secondary main term of size $g^2$ in the second moment using upper bounds for the moments of these *L*-functions on the critical line. Conjectures for the integral moments of this family were explicitly written down by Andrade, Jung and Shamesaldeen in [5].

Our main result extends the study of these moments of Dirichlet *L*-functions over prime polynomials and provides an asymptotic formula for the mixed second moment of their derivatives.

### Theorem 1.1

Let $\mu , \nu \ge 0$ be integers. Then, as $g \rightarrow \infty$,

$$\begin{aligned} \frac{1}{|\mathcal {P}_{2g+1}|} \sum _{P \in \mathcal {P}_{2g+1}} \frac{L^{(\mu )}(\tfrac{1}{2},\chi _P) L^{(\nu )}(\tfrac{1}{2},\chi _P)}{(\log q)^{\mu +\nu }}=\frac{c(\mu ,\nu )}{\zeta _q(2)} \cdot (2g+1)^{\mu +\nu +3}+O(g^{\mu +\nu +2}), \end{aligned}$$

where

1.3 $$\begin{aligned} c(\mu ,\nu )=\frac{1}{2^{\mu +\nu +3}} \left( (-1)^{\mu +\nu } A(\mu ,\nu )+\sum _{m=0}^{\mu } \sum _{n=0}^{\nu } \left( {\begin{array}{c}\mu \\ m\end{array}}\right) \left( {\begin{array}{c}\nu \\ n\end{array}}\right) (-2)^{\mu +\nu -m-n} A(m,n) \right) , \end{aligned}$$

$\zeta _q(s)$ is the zeta-function of $\mathbb {F}_q[t]$, and

1.4 $$\begin{aligned} A(m,n):=\frac{1}{2 (m+n+3)} \int _0^1 \left( x^{m+1} (2-x)^n+x^{n+1} (2-x)^m \right) dx. \end{aligned}$$

The method of proof for Theorem 1.1 involves using an approximate functional equation for the product of two shifted *L*-functions and then taking derivatives of this expression with respect to the shift parameters. Following [7], we then compute the main term by looking at the contribution of the diagonal terms in the resulting sums and bound the error using the Weil bound for character sums over prime polynomials (see Lemma 2.3). As mentioned in [18, Remark 1.3], one could derive a formula for the mixed moments by first computing the shifted moments and then taking derivatives. However, this approach leads to very complicated expressions when the orders of the derivatives are not fixed and small. In the case $\mu =\nu =0$, we recover the asymptotic formula of Andrade and Keating as $c(0,0)=1/24$. It is also possible that one could obtain a secondary main term in Theorem 1.1 using the approach of Bui and Florea in [12].

The study of moments of derivatives of *L*-functions over function fields has already seen a significant amount of interest. Andrade and Yiasemides [9] have obtained asymptotic formulae for the first, second and mixed fourth moment of derivatives of Dirichlet *L*-functions, where the average is over all non-trivial characters modulo a monic, irreducible $Q \in \mathbb {F}_q[t]$. Similarly to the Riemann zeta-function, this family of *L*-functions has unitary symmetry type and as such, one sees a very clear analogy between the results of [9] and those of Conrey [15] on the zeta function.

Andrade and Rajagopal [3] and Andrade and Jung [2] studied the mean values of the derivatives $L^{(n)}(\tfrac{1}{2},\chi _D)$ over the hyperelliptic ensemble $\mathcal {H}_{2g+1}$. Their general formula [2, Theorem 3.1] implies that for any integer $n \ge 1$, as $g \rightarrow \infty$,

1.5 $$\begin{aligned} \frac{1}{|\mathcal {H}_{2g+1}|} \sum _{D \in \mathcal {H}_{2g+1}} \frac{L^{(n)}(\tfrac{1}{2},\chi _D)}{(\log q)^n} \sim \frac{(-1)^n}{2(n+1)} \cdot \mathcal {A}(1) \cdot (2g+1)^{n+1}. \end{aligned}$$

The factor $\mathcal {A}(1)$ is an arithmetic term given in the form of an Euler product which also appears in the asymptotic formula for the first moment of $L(\tfrac{1}{2},\chi _D)$ in [6]. In [10], Bae and Jung used the approach of [21] to obtain lower order terms in the asymptotic formula for the first moment of $L''(\tfrac{1}{2},\chi _D)$ obtained in [3]. It is also shown in [10] that for these quadratic *L*-functions,

$$\begin{aligned} \frac{L'(\tfrac{1}{2},\chi _D)}{-\log q}=g L(\tfrac{1}{2},\chi _D), \end{aligned}$$

and so the moments of $L'(\tfrac{1}{2},\chi _D)$ may be obtained easily from the moments of $L(\tfrac{1}{2},\chi _D)$. In particular, one has all the moments of $L'(\tfrac{1}{2},\chi _D)$ up to the fourth using the results of Florea [19–21].

Djanković and Đokić [18] considered the mixed second moment of $L(s,\chi _D)$ and its second derivative and obtained the asymptotic formula^1^

1.6 $$\begin{aligned} \frac{1}{|\mathcal {H}_{2g+1}|} \sum _{D \in \mathcal {H}_{2g+1}} \frac{L(\tfrac{1}{2},\chi _D) L''(\tfrac{1}{2},\chi _D)}{\log ^2 q} \sim \frac{1}{80} \cdot \frac{\mathcal {B}(1)}{\zeta _q(2)} \cdot (2g+1)^5. \end{aligned}$$

Similarly to (1.5), $\mathcal {B}(1)$ is an arithmetic factor, given as an Euler product, also appearing in the main term of the second moment of $L(\tfrac{1}{2},\chi _D)$ obtained in [20].

The mean values of derivatives of quadratic Dirichlet *L*-functions over monic and irreducible polynomials were first studied by Andrade [1] who obtained an asymptotic formula for the first moment of $L'(\tfrac{1}{2},\chi _P)$ and $L''(\tfrac{1}{2},\chi _P)$. In [25], Jung extended the results of [1] to give an asymptotic formula for the first moment of $L^{(n)}(\tfrac{1}{2},\chi _P)$ over $\mathcal {P}_{2g+1}$ for all integers $n \ge 1$. Jung’s result implies that at as $g \rightarrow \infty$,

1.7 $$\begin{aligned} \frac{1}{|\mathcal {P}_{2g+1}|} \sum _{P \in \mathcal {P}_{2g+1}} \frac{L^{(n)}(\tfrac{1}{2},\chi _P)}{(\log q)^n} \sim \frac{(-1)^n}{2(n+1)} \cdot (2g+1)^{n+1}. \end{aligned}$$

We note the similarity between (1.5) and (1.7) as both families of *L*-functions have symplectic symmetry type. We also include the following result on the twisted first moment of $L^{(k)}(\tfrac{1}{2},\chi _P)$ which is a generalisation of Jung’s result. Before stating the result, we will denote by [*x*] the largest integer that is at most *x* and write $\deg (f)$ for the degree of a polynomial *f*. For any integers $k,n\ge 0$, we let

$$\begin{aligned} J_{k}(n):=\sum _{m=1}^n m^k. \end{aligned}$$

Faulhaber’s formula states that

1.8 $$\begin{aligned} J_{k}(n)=\frac{1}{k+1} \sum _{m=0}^{k} \left( {\begin{array}{c}k+1\\ m\end{array}}\right) B_m^{+} n^{k+1-m}, \end{aligned}$$

where $B_n^{+}$ are the second Bernoulli numbers. In particular, $J_{k}(n)$ is a polynomial in *n* of degree $k+1$ with zero constant term.

### Theorem 1.2

Let $k \ge 0$ be an integer. Also, let $l \in \mathbb {F}_q[t]$ be monic and write $l = l_1 l_2^2$ with $l_1, l_2$ monic and $l_1$ square-free. Then, as $g \rightarrow \infty$,

$$\begin{aligned}&\frac{1}{|\mathcal {P}_{2g+1}|} \sum _{P\in \mathcal {P}_{2g+1}} \frac{L^{(k)}(\tfrac{1}{2},\chi _P) \chi _P(l)}{(\log q)^k}\\&\quad =\frac{(-1)^k}{|l_1|^{1/2}} \sum _{m=0}^k \left( {\begin{array}{c}k\\ m\end{array}}\right) 2^m \deg (l_1)^{k-m} J_m([\tfrac{g-\deg (l_1)}{2}]) \\&\qquad +\frac{1}{|l_1|^{1/2}} \sum _{m=0}^k \left( {\begin{array}{c}k\\ m\end{array}}\right) (-2g)^{k-m} \sum _{i=0}^m \left( {\begin{array}{c}m\\ i\end{array}}\right) 2^i \deg (l_1)^{m-i} J_i([\tfrac{g-1-\deg (l_1)}{2}]) \\&\qquad +O \left( q^{-g/2} g^{k+1} \deg (l) \right) . \end{aligned}$$

Lastly, random matrix theory provides us with predictions for the asymptotic behaviour of the moments of derivatives of the *L*-functions mentioned above. The families of quadratic *L*-functions $L(s,\chi _D)$ and $L(s,\chi _P)$ are examples of families with symplectic symmetry and so we use the ensemble of random unitary symplectic matrices to model the families and formulate conjectures. In [4], Andrade and the author obtain asymptotic formulae for the joint moments of derivatives of the characteristic polynomials of these matrices. They prove that for non-negative integers $k_1, k_2$ and $n_1, n_2$ as the matrix size $N \rightarrow \infty$,

1.9 $$\begin{aligned}  &   \int _{Sp(2N)} \left( \Lambda _A^{(n_1)}(1) \right) ^{k_1} \left( \Lambda _A^{(n_2)}(1) \right) ^{k_2} dA\nonumber \\  &   \quad = b^{Sp}_{k_1, k_2}(n_1, n_2) \cdot (2N)^{k(k+1)/2+k_1 n_2+k_2 n_2} \left( 1+O(N^{-1}) \right) , \end{aligned}$$

where $k=k_1+k_2$. Here, *Sp*(2*N*) denotes the group of $2N \times 2N$ unitary symplectic matrices and *dA* is the Haar measure. Also, the leading order coefficient $b^{Sp}_{k_1, k_2}(n_1, n_2)$ can be written explicitly in the form of a combinatorial sum over partitions (see Theorems 2.1 and 2.2 in [4] for a precise expression). The result in (1.9) allows for conjectures to be made for the corresponding mixed moments of *L*-functions with symplectic symmetry. For instance, for the family $L(s,\chi _P)$, the conjecture is that

$$\begin{aligned} \frac{1}{|\mathcal {P}_{2g+1}|} \sum _{P \in \mathcal {P}_{2g+1}} \frac{L^{(n_1)}(\tfrac{1}{2},\chi _P)^{k_1} L^{(n_2)}(\tfrac{1}{2},\chi _P)^{k_2}}{(\log q)^{n_1+n_2}} \sim \eta _k \cdot b^{Sp}_{k_1, k_2}(n_1, n_2) \cdot (2g+1)^{k(k+1)/2+k_1 n_1+k_2 n_2}, \end{aligned}$$

where $\eta _k$ is a certain arithmetic factor in the form of an Euler product. More specifically, $\eta _k$ is the same arithmetic factor present in the conjectural asymptotic formula for the *k*-th moment of $L(\tfrac{1}{2},\chi _P)$ presented in [5] (see Conjecture 2.2 and Theorem 4.1 in [5] for further details on the conjecture and the arithmetic term). A similar formula is also conjectured to hold for the family $L(s,\chi _D)$ over $\mathcal {H}_{2g+1}$ with a corresponding arithmetic factor. The results of (1.5), (1.6) and (1.7) all agree with the prediction of the conjecture based on random matrix theory since it is shown in [4] that

$$\begin{aligned} b_{0,1}^{Sp}(0,n)=\frac{(-1)^n}{2(n+1)} \ \text {and} \ b_{1,1}^{Sp}(0,2)=\frac{1}{80}. \end{aligned}$$

In regards to the mixed second moment considered in Theorem 1.1, we see that the main term is of the correct size as predicted by the conjecture. The conjecture also states that the factor $c(\mu , \nu )$ in the leading order coefficient should satisfy

1.10 $$\begin{aligned} c(\mu ,\nu )=b_{1,1}^{Sp}(\mu ,\nu ), \end{aligned}$$

since $$\zeta _q(2)^{-1}$$ is the relevant arithmetic factor for the second moment. In this case, the random matrix theory coefficient $$ b_{1,1}^{Sp}(n_1,n_2)$$ has the following explicit expression from [4, Theorem 2.2]:

$$\begin{aligned} b_{1,1}^{Sp}(n_1,n_2)&=\frac{(-1)^{n_1+n_2}}{2^{n_1+n_2+3}} (n_1!) (n_2!) \sum _{2l_1+2l_2 \le n_1} \sum _{2m_1+2m_2 \le n_2} \frac{1}{(n_1-2l_1-2l_2)!} \frac{1}{(n_2-2m_1-2m_2)!} \nonumber \\&\qquad \times \frac{(2l_2+2m_2-2l_1-2m_1-2)}{(2l_1+2m_1+3)! (2l_2+2m_2+1)!}, \end{aligned}$$

where the sum is over non-negative integers $l_1, l_2$ and $m_1, m_2$. We do not attempt to prove that (1.10) holds for all $\mu ,\nu$ here but we have checked numerically that it does indeed hold for $\mu ,\nu \le 20$.

## Background on *L*-functions over function fields

In this section we recall the necessary background on quadratic Dirichlet *L*-functions over function fields. We use [33] as a general reference. Throughout, we let $\mathbb {F}_q$ be a finite field with *q* odd. We denote by $\mathcal {M}$ the set of monic polynomials in $\mathbb {F}_q[t]$ and by $\mathcal {M}_n$ and $\mathcal {M}_{\le n}$ the sets of monic polynomials of degree *n* and of degree at most *n*, respectively. Also, $\mathcal {P}$ denotes the set of monic, irreducible polynomials and $\mathcal {P}_n$ denotes the set of monic, irreducible polynomials of degree *n*. Similarly, $\mathcal {H}$ denotes the set of monic, square-free polynomials and $\mathcal {H}_n$ the set of monic, square-free polynomials of degree *n*. For a polynomial $f \in \mathbb {F}_q[t]$, the norm |*f*| of *f* is defined to be $q^{\deg (f)}$ if $f \ne 0$ and $|f|=0$ if $f=0$. The zeta function $\zeta _q(s)$ of $\mathbb {F}_q[t]$ is defined for $\textrm{Re}(s)>1$ by the Dirichlet series and Euler product

$$\begin{aligned} \zeta _q(s)=\sum _{f \in \mathcal {M}} \frac{1}{|f|^s}=\prod _{P \in \mathcal {P}} \left( 1-\frac{1}{|P|^s} \right) ^{-1}. \end{aligned}$$

As there are $q^n$ monic polynomials of degree *n*, we have that

$$\begin{aligned} \zeta _q(s)=\frac{1}{1-q^{1-s}}. \end{aligned}$$

For an integer $n \ge 1$, the Prime Polynomial Theorem states that

$$\begin{aligned} |\mathcal {P}_n|=\frac{q^n}{n}+O \left( \frac{q^{n/2}}{n} \right) . \end{aligned}$$

Given a monic, irreducible polynomial $P \in \mathcal {P}_{2g+1}$, we define the quadratic character $\chi _P$ using the Legendre symbol

$$\begin{aligned} \chi _P(f)=\left( \frac{f}{P} \right) . \end{aligned}$$

That is,

$$\begin{aligned} \chi _P(f)={\left\{ \begin{array}{ll} 0, &  \text { if } P|f, \\ 1, &  \text { if } P \not \mid f \text { and } f \text { is a square modulo } P, \\ -1, &  \text { if } P \not \mid f \text { and } f \text { is not a square modulo } P. \end{array}\right. } \end{aligned}$$

The quadratic Dirichlet *L*-function attached to the character $\chi _P$ is defined for $Re (s)>1$ by

$$\begin{aligned} L(s,\chi _P)=\sum _{f \in \mathcal {M}} \frac{\chi _P(f)}{|P|^s}=\prod _{Q \in \mathcal {P}} \left( 1-\frac{\chi _P(Q)}{|Q|^s} \right) ^{-1}. \end{aligned}$$

With the change of variables $u=q^{-s}$, we have

$$\begin{aligned} \mathcal {L}(u,\chi _P):=L(s,\chi _P)=\sum _{f \in \mathcal {M}} \chi _P(f) u^{\deg (f)}=\prod _{Q \in \mathcal {P}} \left( 1-\chi _P(Q) u^{\deg (Q)} \right) ^{-1}. \end{aligned}$$

The orthogonality of Dirichlet characters implies that $\mathcal {L}(u,\chi _P)$ is in fact a polynomial in *u* of degree 2*g*. Additionally, it satisfies the functional equation

$$\begin{aligned} \mathcal {L}(u,\chi _P)=(qu^2)^g \mathcal {L} \left( \frac{1}{qu},\chi _P \right) . \end{aligned}$$

By the Riemann Hypothesis for curves over finite fields, proven by Weil [39], all of the zeros of $\mathcal {L}(u,\chi _P)$ lie on the circle $|u|=q^{-1/2}$.

### Preliminary tools

Here we collect all of the necessary tools for the proofs of our main results. First, we denote the divisor function on $\mathbb {F}_q[t]$ by $\tau (f)$ which satisfies

$$\begin{aligned} \sum _{f \in \mathcal {M}_n} \tau (f)=(n+1) q^n, \end{aligned}$$

and for $\alpha ,\beta \in \mathbb {C}$, we let

2.1 $$\begin{aligned} \tau _{\alpha ,\beta }(f)=\sum _{f=f_1 f_2} \frac{1}{|f_1|^{\alpha } |f_2|^{\beta }}. \end{aligned}$$

Also, for integers $m,n \ge 0$ we will denote

$$\begin{aligned} \tau ^{(m,n)}(f):=\frac{\partial ^{m+n}}{\partial \alpha ^m \partial \beta ^n} \tau _{\alpha ,\beta }(f) |_{\alpha =\beta =0}=(-\log q)^{m+n} \sum _{f=f_1 f_2} \deg (f_1)^m \deg (f_2)^n, \end{aligned}$$

and note that we have the bound

$$\begin{aligned} |\tau ^{(m,n)}(f)| \ll \sum _{f=f_1 f_2} \deg (f)^{m+n} \ll \tau (f) \deg (f)^{m+n}. \end{aligned}$$

Next, we have the following ‘approximate’ functional equations which give us exact expressions for both the derivatives of $L(s,\chi _P)$ at $s=1/2$ and the product of two shifted *L*-functions.

#### Lemma 2.1

Let $P \in \mathcal {P}_{2g+1}$. For any integer $\mu \ge 0$, we have

$$\begin{aligned} \frac{L^{(\mu )}(\tfrac{1}{2},\chi _P)}{(\log q)^{\mu }}=\sum _{n=0}^g (-n)^{\mu } q^{-n/2} \sum _{f\in \mathcal {M}_n} \chi _P(f)+\sum _{m=0}^{\mu } \left( {\begin{array}{c}\mu \\ m\end{array}}\right) (-2g)^{\mu -m} \sum _{n=0}^{g-1} n^m q^{-n/2} \sum _{f\in \mathcal {M}_n} \chi _P(f). \end{aligned}$$

#### Proof

This is Lemma 5.1 in [2]. $\square$

#### Lemma 2.2

Let $P \in \mathcal {P}_{2g+1}$. For any $\alpha ,\beta \in \mathbb {C}$, we have

$$\begin{aligned} L(\tfrac{1}{2}+\alpha ,\chi _P) L(\tfrac{1}{2}+\beta ,\chi _P)=\sum _{f\in \mathcal {M}_{\le 2g}} \frac{\tau _{\alpha ,\beta }(f) \chi _P(f)}{|f|^{1/2}}+q^{-2g(\alpha +\beta )} \sum _{f\in \mathcal {M}_{\le 2g-1}} \frac{\tau _{-\alpha ,-\beta }(f) \chi _P(f)}{|f|^{1/2}}, \end{aligned}$$

with $\tau _{\alpha ,\beta }(f)$ defined in (2.1).

#### Proof

This follows from Lemma 2.1 in [13]. $\square$

Lastly, we have the Weil bound for character sums over monic, irreducible polynomials.

#### Lemma 2.3

For $f \in \mathcal {M}$ not a square, as $g \rightarrow \infty$,

$$\begin{aligned} \frac{1}{|\mathcal {P}_{2g+1}|} \sum _{P \in \mathcal {P}_{2g+1}} \chi _P(f) \ll q^{-g} \deg (f). \end{aligned}$$

#### Proof

This follows from equation (2.5) in [34] and the Prime Polynomial Theorem. $\square$

## The twisted first moment of the *k*-th derivative

Here we will prove Theorem 1.2 following the approach used in [25]. Let $l \in \mathcal {M}$ and write $l=l_1 l_2^2$ with $l_1, l_2 \in \mathcal {M}$ and $l_1$ square-free. For $h \in \{g,g-1\}$, we define the sum

$$\begin{aligned} S_h (m;l)&:=\sum _{f \in \mathcal {M}_{\le h}} \frac{\deg (f)^m}{|f|^{1/2}} \frac{1}{|\mathcal {P}_{2g+1}|} \sum _{P\in \mathcal {P}_{2g+1}} \chi _P(fl) \\&=\sum _{n=0}^h n^m q^{-n/2} \sum _{f\in \mathcal {M}_n} \frac{1}{|\mathcal {P}_{2g+1}|} \sum _{P\in \mathcal {P}_{2g+1}} \chi _P(fl), \end{aligned}$$

and compute an asymptotic formula for $S_h (m;l)$ in the following lemma.

### Lemma 3.1

For an integer $m \ge 0$ and $h \in \{g,g-1\}$, we have that as $g \rightarrow \infty$,

$$\begin{aligned} S_h (m;l)=\frac{1}{|l_1|^{1/2}} \sum _{i=0}^m \left( {\begin{array}{c}m\\ i\end{array}}\right) 2^i \deg (l_1)^{m-i} J_i([\tfrac{h-\deg (l_1)}{2}])+O \left( q^{-g/2} g^{m+1} \deg (l) \right) . \end{aligned}$$

### Proof

We split the sum $S_h (m;l)=S_h (m;l)_{\square }+S_h (m;l)_{\ne \square }$ according to whether $fl=\square$ or $fl\ne \square$. For the contribution of non-squares, we use Lemma 2.3 to obtain

$$\begin{aligned} |S_h (m;l)_{\ne \square }|&\ll \sum _{n=0}^g n^m q^{-n/2} \sum _{\begin{array}{c} f\in \mathcal {M}_n \\ fl\ne \square \end{array}} \left| \frac{1}{|\mathcal {P}_{2g+1}|} \sum _{P \in \mathcal {P}_{2g+1}} \chi _P(fl) \right| \\&\ll q^{-g} \sum _{n=0}^g n^m q^{-n/2} \sum _{f\in \mathcal {M}_n} \deg (fl) \\&\ll q^{-g} (g+\deg (l)) \sum _{n=0}^g n^m q^{n/2} \\&\ll q^{-g/2} g^m (g+\deg (l)). \end{aligned}$$

For the contribution of the squares in $S_h (m;l)_{\square }$, we use the facts that $\chi _P(f^2)=\chi _P(f)^2$ and since $\deg (f) \le g$, we have that $P \not \mid f$ for all $P \in \mathcal {P}_{2g+1}$. Thus, for $fl=\square$, we have

$$\begin{aligned} \sum _{P \in \mathcal {P}_{2g+1}} \chi _P(fl)=\sum _{P \in \mathcal {P}_{2g+1}} 1-\sum _{\begin{array}{c} P \in \mathcal {P}_{2g+1}\\ P|l \end{array}} 1=|\mathcal {P}_{2g+1}|+O(\deg (l)). \end{aligned}$$

Now, as $fl=\square$, we write $f=l_1 f_1^2$ with $f_1$ monic. Then, since $\deg (f)=\deg (l_1)+2\deg (f_1) \le h$, we can rewrite the sum over $f_1$ with $\deg (f_1) \le (h-\deg (l_1))/2$ which gives us that

$$\begin{aligned} S_h (m;l)_{\square }&=\sum _{n=0}^h n^m q^{-n/2} \sum _{\begin{array}{c} f\in \mathcal {M}_n \\ fl=\square \end{array}} \frac{1}{|\mathcal {P}_{2g+1}|} \sum _{P \in \mathcal {P}_{2g+1}} \chi _P(fl) \\&=\sum _{n=0}^h n^m q^{-n/2} \sum _{\begin{array}{c} f \in \mathcal {M}_n \\ fl=\square \end{array}} 1+O\left( \frac{\deg (l)}{|\mathcal {P}_{2g+1}|} \sum _{n=0}^h n^m q^{-n/2} \sum _{\begin{array}{c} f\in \mathcal {M}_n \\ fl=\square \end{array}} 1 \right) \\&=q^{-\deg (l_1)/2} \sum _{n=0}^{(h-\deg (l_1))/2} (\deg (l_1)+2n)^m q^{-n} \sum _{f_1 \in \mathcal {M}_n} 1\\&\quad +O\left( q^{-2g} g \, \deg (l) \sum _{n=0}^h n^m q^{n/2} \right) \\&=\frac{1}{|l_1|^{1/2}} \sum _{n=0}^{(h-\deg (l_1))/2} (\deg (l_1)+2n)^m+O\left( q^{-3g/2} g^{m+1} \deg (l) \right) , \end{aligned}$$

where we have used the Prime Polynomial Theorem in bounding the error. For the main term, we use a binomial expansion and the function $J_i(n)=\sum _{m=0}^n m^i$ to write it as

$$\begin{aligned} \frac{1}{|l_1|^{1/2}} \sum _{n=0}^{(h-\deg (l_1))/2} (2n+\deg (l_1))^m&=\frac{1}{|l_1|^{1/2}} \sum _{n=0}^{(h-\deg (l_1))/2} \sum _{i=0}^m \left( {\begin{array}{c}m\\ i\end{array}}\right) (2n)^i \deg (l_1)^{m-i} \\&=\frac{1}{|l_1|^{1/2}} \sum _{i=0}^m \left( {\begin{array}{c}m\\ i\end{array}}\right) 2^i \deg (l_1)^{m-i} \sum _{n=0}^{(h-\deg (l_1))/2} n^i \\&=\frac{1}{|l_1|^{1/2}} \sum _{i=0}^m \left( {\begin{array}{c}m\\ i\end{array}}\right) 2^i \deg (l_1)^{m-i} J_i([\tfrac{h-\deg (l_1)}{2}]). \end{aligned}$$

Combining this with the bounds for the error terms completes the proof. $\square$

### Proof of Theorem 1.2

Using the expression for $L^{(k)}(\tfrac{1}{2},\chi _P)$ given in Lemma 2.1 and multiplying by $\chi _P(l)$, we have that

$$\begin{aligned} \frac{L^{(k)}(\tfrac{1}{2},\chi _P) \chi _P(l)}{(\log q)^k}&=(-1)^k \sum _{n=0}^g n^k q^{-n/2} \sum _{f\in \mathcal {M}_n} \chi _P(fl)\\&\quad +\sum _{m=0}^k \left( {\begin{array}{c}k\\ m\end{array}}\right) (-2g)^{k-m} \sum _{n=0}^{g-1} n^m q^{-n/2} \sum _{f\in \mathcal {M}_n} \chi _P(fl). \end{aligned}$$

Taking the average over $\mathcal {P}_{2g+1}$, we can then write

$$\begin{aligned} \frac{1}{|\mathcal {P}_{2g+1}|} \sum _{P \in \mathcal {P}_{2g+1}} \frac{L^{(k)}(\tfrac{1}{2},\chi _P) \chi _P(l)}{(\log q)^k}=(-1)^k S_g (k;l)+\sum _{m=0}^k \left( {\begin{array}{c}k\\ m\end{array}}\right) (-2g)^{k-m} S_{g-1} (m;l). \end{aligned}$$

Using this expression for the twisted moment and applying the formula for $S_h (m;l)$ in Lemma 3.1 completes the proof. $\square$

## The mixed second moment of the $\mu$-th and $\nu$-th derivatives

In this section will prove Theorem 1.1. First, for integers $m,n \ge 0$ and $h \in \{2g,2g-1\}$, let

4.1 $$\begin{aligned} T_h(m,n)=\sum _{f\in \mathcal {M}_{\le h}} \frac{\tau ^{(m,n)}(f)}{|f|^{1/2}} \frac{1}{|\mathcal {P}_{2g+1}|} \sum _{P\in \mathcal {P}_{2g+1}} \chi _P(f). \end{aligned}$$

The following lemma establishes the asymptotic behaviour of the sums $T_h(m,n)$ as $g \rightarrow \infty$.

### Lemma 4.1

For integers $m,n \ge 0$ and $h \in \{2g, 2g-1\}$, we have that as $g \rightarrow \infty$,

4.2 $$\begin{aligned} T_h(m,n)=\frac{(-\log q)^{m+n} A(m,n)}{\zeta _q(2)} g^{m+n+3}+O(g^{m+n+2}), \end{aligned}$$

with *A*(*m*, *n*) as defined in (1.4).

### Proof

We write

$$\begin{aligned} T_h(m,n)=T_h(m,n)_{\square }+T_h(m,n)_{\ne \square }, \end{aligned}$$

where $T_h(m,n)_{\square }$ and $T_h(m,n)_{\ne \square }$ denote the sums over *f* a perfect square and *f* not a square, respectively. For the terms with $f \ne \square$, we bound the sum using Lemma 2.3 to obtain

$$\begin{aligned} |T_h(m,n)_{\ne \square }|&\ll \sum _{\begin{array}{c} f\in \mathcal {M}_{\le 2g}\\ f\ne \square \end{array}} \frac{\tau ^{(m,n)}(f)}{|f|^{1/2}} \left| \frac{1}{|\mathcal {P}_{2g+1}|} \sum _{P\in \mathcal {P}_{2g+1}} \chi _P(f) \right| \\&\ll q^{-g} \sum _{f\in \mathcal {M}_{\le 2g}} \frac{\tau ^{(m,n)}(f)}{|f|^{1/2}} \deg (f) \\&\ll g q^{-g} \sum _{j=0}^{2g} q^{-j/2} \sum _{f\in \mathcal {M}_j} \tau (f) \deg (f)^{m+n} \\&\ll g q^{-g} \sum _{j=0}^{2g} j^{m+n+1} q^{j/2} \\&\ll g^{m+n+2}, \end{aligned}$$

where we have used the fact that $\sum _{f\in \mathcal {M}_j} \tau (f)\ll j q^j$. For the terms in $T_h(m,n)_{\square }$ with $f=\square$, we have that

$$\begin{aligned} \frac{1}{|\mathcal {P}_{2g+1}|} \sum _{P\in \mathcal {P}_{2g+1}} \chi _P(f)=1, \end{aligned}$$

since $\deg (f) \le 2g$ and so $P \not \mid f$ for all $P \in \mathcal {P}_{2g+1}$. Thus, we have that

$$\begin{aligned} T_h(m,n)_{\square }=\sum _{\begin{array}{c} f\in \mathcal {M}_{\le h}\\ f=\square \end{array}} \frac{\tau ^{(m,n)}(f)}{|f|^{1/2}}=\sum _{f\in \mathcal {M}_{\le h/2}} \frac{\tau ^{(m,n)}(f^2)}{|f|}. \end{aligned}$$

We next write the main term $T_h(m,n)_{\square }$ as

$$\begin{aligned} \sum _{f\in \mathcal {M}_{\le h/2}} \frac{\tau ^{(m,n)}(f^2)}{|f|}=(-\log q)^{m+n} \sum _{j=0}^{h/2} q^{-j} \sum _{f \in \mathcal {M}_j} \sum _{f^2=f_1 f_2} \deg (f_1)^m \deg (f_2)^n. \end{aligned}$$

Then, for a given *j*, we use the hyperbola method and write

4.3 $$\begin{aligned} \sum _{f \in \mathcal {M}_j} \sum _{f^2=f_1 f_2} \deg (f_1)^m \deg (f_2)^n&=\sum _{f \in \mathcal {M}_j} \sum _{\begin{array}{c} f^2=f_1 f_2 \\ \deg (f_1)=j \end{array}} j^{m+n}+\sum _{f \in \mathcal {M}_j} \sum _{\begin{array}{c} f^2=f_1 f_2 \\ \deg (f_1)<j \end{array}} \deg (f_1)^m \deg (f_2)^n \nonumber \\&\qquad +\sum _{f \in \mathcal {M}_j} \sum _{\begin{array}{c} f^2=f_1 f_2 \\ \deg (f_2)<j \end{array}} \deg (f_1)^m \deg (f_2)^n. \end{aligned}$$

The second and third sums here are similar so we need only focus on the first two. In particular, it suffices to evaluate the sum

4.4 $$\begin{aligned} \sum _{f \in \mathcal {M}_j} \sum _{\begin{array}{c} f^2=f_1 f_2 \\ \deg (f_1)=k \end{array}} \deg (f_1)^m \deg (f_2)^n=k^m (2j-k)^n \sum _{f \in \mathcal {M}_j} \sum _{\begin{array}{c} f^2=f_1 f_2 \\ \deg (f_1)=k \end{array}} 1, \end{aligned}$$

for $k \le j$. To compute the above sum, we observe that $f_1 f_2=\square$ if and only if $f_1=l_1 l_2^2$ and $f_2=l_1 l_3^2$ with $l_1, l_2, l_3 \in \mathcal {M}$ and $l_1$ square-free. Also, since $\deg (f_1)=k$, we must have $\deg (l_1) \le k$ and $\deg (l_2)=(k-\deg (l_1))/2$. Then, as $\deg (f^2)=\deg (f_1)+\deg (f_2)=2j$, we have that $\deg (l_3)=j-k/2-\deg (l_1)/2 \ge 0$. So, by summing over $l_1, l_2$ and $l_3$, we have that

$$\begin{aligned} \sum _{f \in \mathcal {M}_j} \sum _{\begin{array}{c} f^2=f_1 f_2 \\ \deg (f_1)=k \end{array}} 1&=\sum _{\begin{array}{c} l_1 \in \mathcal {H} \\ \deg (l_1) \le k \\ k-\deg (l_1) \ even \end{array}} \sum _{\begin{array}{c} l_2 \in \mathcal {M} \\ \deg (l_2)=(k-\deg (l_1))/2 \end{array}} \sum _{\begin{array}{c} l_3 \in \mathcal {M} \\ \deg (l_3)=j-(k+\deg (l_1))/2 \end{array}} 1 \\&=q^{j-k/2} \sum _{\begin{array}{c} l_1 \in \mathcal {H} \\ \deg (l_1) \le k \\ k-\deg (l_1) \ even \end{array}} q^{-\deg (l_1)/2} \sum _{\begin{array}{c} l_2 \in \mathcal {M} \\ \deg (l_2)=(k-\deg (l_1))/2 \end{array}} 1 \\&=q^j \sum _{\begin{array}{c} l_1 \in \mathcal {H} \\ \deg (l_1) \le k \\ k-\deg (l_1) \ \text {even} \end{array}} q^{-\deg (l_1)}. \end{aligned}$$

For the final sum over $l_1$, if *k* is even, then

$$\begin{aligned} \sum _{\begin{array}{c} l_1 \in \mathcal {H} \\ \deg (l_1) \le k \\ k-\deg (l_1) \ even \end{array}} q^{-\deg (l_1)}=\sum _{\begin{array}{c} i=0 \\ i \ even \end{array}}^k \sum _{l_1 \in \mathcal {H}_i} q^{-i}=1+\sum _{i=1}^{k/2} \sum _{l_1 \in \mathcal {H}_{2i}} q^{-2i}=1+(1-q^{-1}) \frac{k}{2}, \end{aligned}$$

where we have used the fact that for $n \ge 1$,

$$\begin{aligned} |\mathcal {H}_n|=\frac{q^n}{\zeta _q(2)}=q^n (1-q^{-1}). \end{aligned}$$

Similarly, if *k* is odd, we have

$$\begin{aligned} \sum _{\begin{array}{c} l_1 \in \mathcal {H} \\ \deg (l_1) \le k \\ k-\deg (l_1) \ even \end{array}} q^{-\deg (l_1)}=1+(1-q^{-1}) \frac{k-1}{2}. \end{aligned}$$

Thus, we have that

$$\begin{aligned} \sum _{f \in \mathcal {M}_j} \sum _{\begin{array}{c} f^2=f_1 f_2 \\ \deg (f_1)=k \end{array}} 1=q^j \left( 1+(1-q^{-1}) [k/2] \right) , \end{aligned}$$

and incorporating this into (4.3) and (4.4) gives us

4.5 $$\begin{aligned}&\sum _{f \in \mathcal {M}_j} \sum _{f^2=f_1 f_2} \deg (f_1)^m \deg (f_2)^n \nonumber \\&=q^j \left( j^{m+n} \left( 1+(1-q^{-1}) [j/2] \right) +\sum _{k=0}^{j-1} \left( k^m (2j-k)^n+k^n (2j-k)^m \right) \left( 1+(1-q^{-1}) [k/2] \right) \right) . \end{aligned}$$

We now approximate the expression in (4.5) as $j \rightarrow \infty$. As

$$\begin{aligned} \left( 1+(1-q^{-1}) [k/2] \right) =(1-q^{-1}) \frac{k}{2}+O(1), \end{aligned}$$

the first term in the brackets in (4.5) is

$$\begin{aligned} (1-q^{-1}) \frac{j^{m+n+1}}{2}+O(j^{m+n}). \end{aligned}$$

Next, we have that

4.6 $$\begin{aligned} \sum _{k=0}^{j-1} k^m (2j-k)^n \left( 1+(1-q^{-1}) [k/2] \right)&=\frac{(1-q^{-1})}{2} \sum _{k=0}^{j-1} k^{m+1} (2j-k)^n\nonumber \\&\quad +O \left( \sum _{k=0}^{j-1} k^m (2j-k)^n \right) , \end{aligned}$$

so we now focus on the sum $\sum _{k=0}^{j-1} k^m (2j-k)^n$. On the one hand, by writing it in terms of a Riemann sum, we have

$$\begin{aligned} \sum _{k=0}^{j-1} k^m (2j-k)^n=j^{m+n+1} \cdot \frac{1}{j} \sum _{k=0}^{j-1} \left( \frac{k}{j} \right) ^m \left( 2-\frac{k}{j} \right) ^n \sim j^{m+n+1} \int _0^1 x^m (2-x)^n dx, \end{aligned}$$

as $j \rightarrow \infty$. On the other hand, by Faulhaber’s formula, we know that the sum is a polynomial in *j*. Thus, we have that

4.7 $$\begin{aligned} \sum _{k=0}^{j-1} k^m (2j-k)^n=j^{m+n+1} \int _0^1 x^m (2-x)^n dx+O(j^{m+n}), \end{aligned}$$

and then using (4.6) and (4.7) in (4.5) yields

$$\begin{aligned} \sum _{f \in \mathcal {M}_j} \sum _{f^2=f_1 f_2} \deg (f_1)^m \deg (f_2)^n&=\frac{q^j j^{m+n+2}}{2 \zeta _q(2)} \int _0^1 \left( x^{m+1} (2-x)^n+x^{n+1} (2-x)^m \right) dx\\&\quad +O(q^j j^{m+n+1}). \end{aligned}$$

Therefore, by Faulhaber’s formula (1.8),

$$\begin{aligned}&\sum _{f \in \mathcal {M}_{\le h/2}} \frac{\tau ^{(m,n)}(f^2)}{|f|} \\&\quad =(-\log q)^{m+n} \sum _{j=0}^{h/2} q^{-j} \sum _{f \in \mathcal {M}_j} \sum _{f^2=f_1 f_2} \deg (f_1)^m \deg (f_2)^n \\&\quad =\frac{(-\log q)^{m+n}}{2 \zeta _q(2)} \int _0^1 \left( x^{m+1} (2-x)^n+x^{n+1} (2-x)^m \right) dx \sum _{j=0}^{h/2} j^{m+n+2}\\&\qquad +O \left( \sum _{j=0}^{h/2} j^{m+n+1} \right) \\&\quad =\frac{(-\log q)^{m+n}}{2^{m+n+4} (m+n+3) \zeta _q(2)} \cdot h^{m+n+3} \int _0^1 \left( x^{m+1} (2-x)^n+x^{n+1} (2-x)^m \right) dx\\&\qquad +O(h^{m+n+2}). \end{aligned}$$

Recalling the definition of *A*(*m*, *n*) and choosing $h \in \{2g,2g-1\}$ completes the proof. $\square$

We are now ready to prove Theorem 1.1.

### Proof of Theorem 1.1

We begin with the approximate functional equation for the product of two shifted *L*-functions given in Lemma 2.2. For $P \in \mathcal {P}_{2g+1}$, we have that

$$\begin{aligned} L(\tfrac{1}{2}+\alpha ,\chi _P) L(\tfrac{1}{2}+\beta ,\chi _P)=\sum _{f\in \mathcal {M}_{\le 2g}} \frac{\tau _{\alpha ,\beta }(f) \chi _P(f)}{|f|^{1/2}}+q^{-2g(\alpha +\beta )} \sum _{f\in \mathcal {M}_{\le 2g-1}} \frac{\tau _{-\alpha ,-\beta }(f) \chi _P(f)}{|f|^{1/2}}. \end{aligned}$$

Using the approximate functional equation, we write

4.8 $$\begin{aligned} \frac{1}{|\mathcal {P}_{2g+1}|} \sum _{P \in \mathcal {P}_{2g+1}} L(\tfrac{1}{2}+\alpha ,\chi _P) L(\tfrac{1}{2}+\beta ,\chi _P)=F_{2g}(\alpha ,\beta )+q^{-2g(\alpha +\beta )} F_{2g-1}(-\alpha ,-\beta ), \end{aligned}$$

where for $h \in \{2g,2g-1\}$, we define

$$\begin{aligned} F_{h}(\alpha ,\beta ):=\sum _{f\in \mathcal {M}_{\le h}} \frac{\tau _{\alpha ,\beta }(f)}{|f|^{1/2}} \frac{1}{|\mathcal {P}_{2g+1}|} \sum _{P\in \mathcal {P}_{2g+1}} \chi _P(f). \end{aligned}$$

For integers $m,n \ge 0$, we then have that

$$\begin{aligned} \frac{\partial ^{m+n}}{\partial \alpha ^m \partial \beta ^n} F_h(\alpha ,\beta ) |_{\alpha =\beta =0}=T_h(m,n)=\sum _{f\in \mathcal {M}_{\le h}} \frac{\tau ^{(m,n)}(f)}{|f|^{1/2}} \frac{1}{|\mathcal {P}_{2g+1}|} \sum _{P\in \mathcal {P}_{2g+1}} \chi _P(f). \end{aligned}$$

Thus, differentiating (4.8) with respect to $\alpha$ and $\beta$ and setting $\alpha =\beta =0$ leads to

$$\begin{aligned}&\frac{1}{|\mathcal {P}_{2g+1}|} \sum _{P \in \mathcal {P}_{2g+1}} L^{(\mu )}(\tfrac{1}{2},\chi _P) L^{(\nu )}(\tfrac{1}{2},\chi _P) \nonumber \\&\qquad =T_{2g}(\mu ,\nu )+(-1)^{\mu +\nu } \sum _{m=0}^{\mu } \sum _{n=0}^{\nu } \left( {\begin{array}{c}\mu \\ m\end{array}}\right) \left( {\begin{array}{c}\nu \\ n\end{array}}\right) (2g \log q)^{\mu +\nu -m-n} \, T_{2g-1}(m,n). \end{aligned}$$

Theorem 1.1 then follows on applying the asymptotic formula for $T_h(m,n)$ in Lemma 4.1. $\square$

## Data Availability

Data sharing is not applicable to this article as no datasets were generated or analysed for this work.

## Footnotes

1 In [18], the main result is given in terms of the completed *L*-function rather than $L(s,\chi _D)$ but, as discussed in [18, Remark 1.2], the formula given in (1.6) follows from [18, Theorem 1.1] and Florea’s formula for the second moment of $L(\tfrac{1}{2},\chi _D)$ in [20].
